# Distinct Functions of Neutrophil in Cancer and Its Regulation

**DOI:** 10.1155/2015/701067

**Published:** 2015-11-16

**Authors:** Zvi Granot, Jadwiga Jablonska

**Affiliations:** ^1^Department of Developmental Biology and Cancer Research, The Institute for Medical Research Israel-Canada, The Hebrew University-Hadassah Medical School, 91120 Jerusalem, Israel; ^2^Translational Oncology, Internal Medicine II, University Hospital, Tuebingen, Otfried-Mueller-Street 10, 72076 Tuebingen, Germany

## Abstract

Neutrophils are the most abundant of all white blood cells in the human circulation and are usually associated with inflammation and with fighting infections. In recent years the role immune cells play in cancer has been a matter of increasing interest. In this context the function of neutrophils is controversial as neutrophils were shown to possess both tumor promoting and tumor limiting properties. Here we provide an up-to-date review of the pro- and antitumor properties neutrophils possess as well as the environmental cues that regulate these distinct functions.

## 1. Introduction

Neutrophils are the most abundant of all white blood cells and play a key role in host protection against microbial infections and in inflammation. Chronic inflammation has been associated with increased susceptibility for cancer. Hepatitis B [1] and inflammatory bowel disease [2] are common examples for this correlation, leading to hepatocellular carcinoma and colorectal cancer, respectively. Neutrophils, as a key component in inflammation, may play a crucial role in inflammation driven tumorigenesis. This was well exemplified when neutrophils were shown to directly promote carcinogenesis in a mouse model of colitis [3]. Indeed, neutrophils at the primary tumor site were shown to provide a wide range of different tumor promoting functions. Neutrophils were shown to support angiogenesis via secretion of proangiogenic factors as well as the proteolytic activation of proangiogenic factors. Neutrophils were also implicated in promoting tumor growth through the proteolytic release of EGF, TGF*β*, and PDGF from the extracellular matrix (ECM). Neutrophils express high levels of metalloproteinases which can also modify the ECM to allow tumor cell dissemination thereby promoting tumor spread. Furthermore, neutrophils were shown to recruit other tumor promoting cells to the tumor bed. Finally, immature neutrophils, also termed G-MDSC (granulocytic myeloid derived suppressor cells), were implicated in the establishment of an immunosuppressive tumor microenvironment thereby limiting antitumor immunity. On the other hand, neutrophils were shown to have antitumor properties including the capacity to kill tumor cells either through direct cytotoxicity or via antibody dependent cell cytotoxicity (ADCC) [4]. Similar conflicting reports were made as to the role neutrophils play in the premetastatic niche. Neutrophils accumulate in large numbers in premetastatic organs [5–7]. The fact that bone marrow derived cells were implicated in priming of the premetastatic niche prompted the hypothesis that neutrophils may be the cells that mediate this process. Indeed, neutrophils were shown to have a positive effect on tumor cell seeding in the premetastatic site [6]. In contrast, we and others have shown that neutrophils actively limit metastatic seeding by killing tumor cells [5, 7].

Interestingly, while neutrophils play a role in modulating tumor cell seeding in the metastatic site, it seems like they do not affect the growth rate of the metastatic nodules [5, 7]. This suggested that neutrophil antitumor functions are not always manifested inside the tumor and may depend on the chemokine landscape in the tumor microenvironment. This notion was further supported by findings showing that upon entering the tumor microenvironment neutrophils acquire a different set of traits. This was referred to as “polarization” of neutrophils toward a tumor promoting or an antitumor phenotype which is mediated via cytokines available in the tumor microenvironment (i.e., TGF*β* and IFNs, resp.). Furthermore, recent studies suggested that neutrophils are not a homogeneous population of cells and may consist of both pro- and antitumor subpopulations [8]. Together, the observations made thus far suggest that the mere accumulation of neutrophils in the tumor site may not necessarily be indicative of their contribution or of their prognostic value. Along these lines, the ongoing efforts to correlate neutrophil counts, or the ratio between neutrophils and other immune cells, with patient prognosis and ultimate outcome are conflicting and show that neutrophil abundance may correlate with a better prognosis in some studies and with a worse prognosis in others [9].

## 2. Molecular Mechanisms of Neutrophil Polarization in the Tumor Microenvironment

Neutrophils were shown to have diverse functions in the tumor microenvironment including both promoting and inhibiting tumor growth. As neutrophils are quick to respond to environmental cues, the most plausible explanation for the different neutrophil phenotypes was that neutrophil function is dictated by the local chemokine milieu. Advances in our understanding of how neutrophil function is regulated in cancer have led to the realization that neutrophils may be directed towards a specific phenotype, be it tumor promoting or tumor limiting, upon entering the tumor. Here we will discuss how interferons and TGF*β* polarize neutrophils in the tumor microenvironment.

### 2.1. Interferons

Type I interferons (IFNs) were first characterized in the process of viral interference. However, since then IFNs were found to be involved in a wide range of biological processes. In the context of cancer, IFNs show strong antitumor function as they inhibit tumor cell proliferation and promote apoptosis [10]. However, IFNs were also found to play a key role in mounting an antitumor immune response through the activation of T-cells, NK cells, and macrophages [11]. In recent years it has become apparent that IFNs also affect neutrophil function and promote antitumor processes mediated by neutrophils. Jablonska et al. have shown that IFN-*β* is critical for suppressing the expression of proangiogenic factors, such as VEGF and MMP9, in tumor infiltrating neutrophils leading to enhanced tumor vascularization and growth in IFN-*β* deficient animals [12]. Furthermore, IFN-*β* was found to play a significant role in regulating the recruitment of neutrophils and their longevity in the primary tumor [13, 14]. Finally, type I IFN activity was found to inhibit neutrophil-mediated formation of “fertile” premetastatic niche [15].

### 2.2. TGF*β*

TGF*β* is a multipotent molecule known to have diverse effects in cancer. One of the most explored functions of TGF*β* in cancer is its role in generating an immunosuppressive tumor microenvironment. A groundbreaking study by Fridlender and colleagues [16] demonstrated that TGF*β* plays a critical role in suppression of antitumor neutrophil cytotoxicity. In this study, the authors showed that blocking TGF*β* signaling leads to a change in the cellular composition of the tumor and allows the influx of large numbers of neutrophils. More importantly, they showed that tumor-associated neutrophils (TANs) recruited in the absence of TGF*β* signaling have an antitumor N1 phenotype. The authors concluded that TGF*β* in the tumor microenvironment is involved in polarizing TAN towards N2 protumor phenotype. This concept was supported by other studies showing that TGF*β* can directly block antitumor neutrophil cytotoxicity [5] and that TGF*β* receptor deficient myeloid cells, including neutrophils, maintain an antitumor phenotype and limit tumor growth [17].

**Figure 1:**
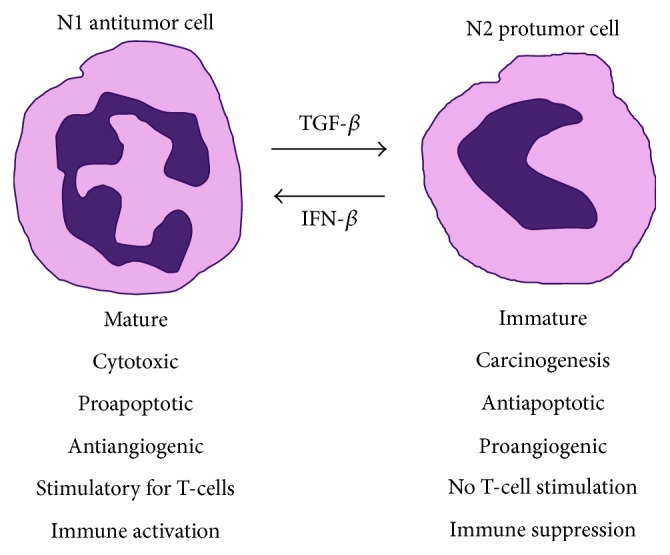
Neutrophil function in cancer is dictated by environmental cues. Neutrophils may be divided into N1 antitumor and N2 protumor cells. TGF*β* is a potent driver of the transition from N1 to N2 phenotype whereas IFN-*β* is a potent driver of the transition in the opposite direction. This exemplifies the notion that neutrophil function in cancer is determined by the chemokine milieu in the microenvironment.

The conflicting effects of TGF*β* and IFNs on neutrophil function in the context of cancer are an example of how neutrophils respond to cues in the microenvironment (Figure 1). While understanding the mechanisms that regulate neutrophil function is clearly important from a therapeutic point of view, the realization that neutrophils may play conflicting roles, depending on their context, is an important notion.

## 3. Antitumor N1 Phenotype

Antitumor N1 neutrophils act to limit tumor growth and metastatic progression. This is accomplished via distinct mechanisms including direct and antibody dependent cytotoxicity as well as through the activation of other cell types including T-cells and dendritic cells.

### 3.1. Direct Cytotoxicity

Direct cytotoxicity of neutrophils towards tumor cells is not a novel concept and was first observed in the early 1970s [18]. Neutrophils are highly motile phagocytic cells whose primary function is antimicrobial protection of the host. Accordingly, neutrophils generate a variety of antimicrobial molecules. However, most of these molecules are harmless to eukaryotic cells. Still, the reactive molecules generate by the NADPH oxidase complex, superoxides, H_2_O_2_, and HOCl. Indeed, these molecules were found to be directly involved in antitumor neutrophil cytotoxicity [19–21]. Several studies have shown that physical contact is required for neutrophil cytotoxicity. However, stimulating cultured neutrophils with a potent agonist, such as PMA, leads to the generation and secretion of very high levels of H_2_O_2_ alleviating the need for physical contact [5].

### 3.2. ADCC

Antibody dependent cell-mediated cytotoxicity (ADCC) is another mechanism for neutrophil antitumor cytotoxicity. Tumor cell-specific antibodies may be successfully used as an anticancer therapy. Antibody labeled cells are susceptible to destruction by immune cells expressing Fc receptors (FcR). Neutrophils express several FcRs that can mediate ADCC including Fc*γ*RI (CD64), Fc*γ*RIIa (CD32), Fc*γ*RIIIa (CD16a), and Fc*γ*RIIIb (CD16b) [22–24]. Indeed, neutrophils were shown to take part in ADCC in several types of cancer including glioma, squamous cell, and ovarian carcinoma. Neutrophils were also shown to contribute to the antitumor ADCC in Non-Hodgkin's Lymphoma [25], in breast cancer using [26], and in B-cell lymphoma [27].

### 3.3. Stimulation of T-Cells and DCs

Neutrophils, on top of having a role in killing tumor cells directly, can also stimulate adaptive antitumor immune responses. This was well exemplified by experiments showing that neutrophils are required for proper antitumor CD8^+^ T-cell immune response [16, 28–30]. Stimulation of adaptive antitumor immunity by neutrophils has two arms, the recruitment of other immune cells and their antigen presenting abilities.

*(a) Recruitment of Immune Cells*. Neutrophils secrete several cytokines including TNF*α*, Cathepsin G, and neutrophil elastase which have a direct effect on T-cells and promote their proliferation and cytokine production. Neutrophils, under these conditions, act to recruit and activate T-cells and enhance the overall adaptive immune antitumor response. Specifically, TAN were shown to stimulate T-cell proliferation and IFN*γ* secretion in early stage lung cancer patients [31].

*(b) Neutrophil Extracellular Traps (NETs)*. Production of extracellular traps by neutrophils is an interesting feature in neutrophil biology. These NETs are composed of chromatin fibers decorated with histones and other proteins and are considered as an additional tool in neutrophils' arsenal of antimicrobial properties. However, Tillack and colleagues showed that NETs may also be utilized to prime T-cells [32]. This was also linked to a possible role of NETs in immunoediting in cancer and the propagation of antitumor immune responses [33]. 

*(c) Antigen Presentation*. For a long time antigen presentation was thought to be exclusively mediated by macrophages and more so by dendritic cells (DCs). However, in 2007 Beauvillain and colleagues demonstrated that neutrophils can efficiently process and present antigens to directly stimulate T-cell immune responses [34]. While this does not directly link neutrophil presentation of antigens to antitumor cytotoxicity, Fridlender and colleagues showed in 2009 that N1 TANs require T-cells for their antitumor activity in the primary tumor [16], an observation that may be explained by neutrophils' ability to present tumor antigens to stimulate T-cells.

## 4. Protumor N2 Phenotype

Neutrophils have been traditionally considered as guards of the host immune system. However, in the context of tumor, the function of these cells is frequently modified to act against the host and promote tumor growth and metastasis formation. A possible reason for this could be tumor-secreted factors that elicit wound-repair responses by neutrophils that in turn inadvertently stimulate tumor progression [35]. Moreover, wound-infiltrating neutrophils are rapidly diverted from a wound to preneoplastic cells and such interactions lead to increased proliferation of the preneoplastic cells. Prostaglandin E2 (PGE2) seems to be the factor responsible for this process [36]. These results have shown that repeated wounding with subsequent inflammation leads to a greater incidence of local melanoma formation. Along these lines, several studies have shown that infiltration of tumors by neutrophils is associated with poor clinical outcome. Tumor-associated neutrophils (TANs) have been shown to promote tumor growth and progression via a variety of mechanisms, including extracellular matrix remodeling, promotion of tumor cell invasion and metastasis, angiogenesis, lymphangiogenesis, and immune suppression [12, 13, 15].

### 4.1. Protumor Cytokines

One of the mechanisms responsible for neutrophil-mediated tumor angiogenesis, growth, and metastasis is the secretion of protumor cytokines by these cells [37]. Depending on the cytokine milieu, neutrophils are able to secrete multiple growth factors such as EGF, TGF*β*, PDGF, HGF, VEGF, and oncostatin M [12, 38–41].

Evidence suggests that EGF and its receptor EGFR are involved in the pathogenesis and progression of different carcinoma types [42]. Amplification of the EGFR gene and mutations of the EGFR tyrosine kinase domain have been recently demonstrated to occur in carcinoma patients. EGFR causes neoangiogenesis but also increased proliferation, decreased apoptosis, and enhanced tumor cell motility [43] since its receptor (EGFR; HER1; erbB1) is highly expressed on variety of human tumors including non-small cell lung cancer (NSCLC) and breast, head and neck, gastric, colorectal, esophageal, prostate, bladder, renal, pancreatic, and ovarian cancers [42, 44].

TGF*β* is frequently upregulated in human cancers [45] and has been linked to the regulation of tumor initiation, progression, and metastasis [46]. Tumor-secreted TGF*β* is usually sequestered to the extracellular matrix as an inactive complex and becomes activated through enzymes such as neutrophil-derived elastase and MMP9 [46]. Furthermore, reactive oxygen free radicals produced by activated neutrophils can activate latent TGF*β* [47]. Thus, activated neutrophils, through production of elastase, MMP9, and ROS, may contribute to TGF*β*-mediated immunosuppression [9]. Furthermore, TGF*β* has been shown to be a potent chemoattractant for neutrophils facilitating their recruitment to sites of inflammation [48, 49] and to promote their protumor N2 phenotype, as mentioned above [16].

Another important neutrophil-derived growth factor is platelet-derived growth factor (PDGF). Interestingly, this growth factor was shown to be chemotactic for monocytes and neutrophils [50]. It was recently established that PDGF stimulation cooperates with genetic changes caused by retroviral insertions in induction of fully malignant tumor phenotype [51]. Moreover, the autocrine PDGF signaling seems to play a role in the growth and metastasis of epithelial cancers.

VEGF is a very potent proangiogenic factor but also serves as a potent chemoattractant for neutrophils. It has been implicated as the key endothelial cell-specific factor required for pathological angiogenesis, including tumor neovascularization. Inhibition of the VEGF signaling blocks angiogenesis in growing tumors, leading to regression of tumor growth [52]. The function of vascular endothelial growth factor (VEGF) in cancer is not limited to angiogenesis and vascular permeability [53]. VEGF-mediated signaling occurs in tumor cells, and this signaling contributes to key aspects of tumorigenesis, including the function of cancer stem cells and tumor initiation [54]. Autocrine VEGF signaling can promote the growth, survival, migration, and invasion of cancer cells [55–57].

Oncostatin M is another pleiotropic cytokine that is secreted by neutrophils [58]. It has been shown to exert proinflammatory effects by inducing adhesion and chemotaxis of neutrophils and chemokine production by endothelial cells [59]. Although oncostatin M was originally identified as an inhibitor of tumor cell growth* in vitro* [60, 61], it is increasingly apparent that this cytokine plays a role in breast cancer cell detachment [62] and angiogenesis [41].

In addition to growth factors, neutrophils are able to secrete other cytokines that influence tumor development and spreading. For instance, neutrophil delivered TNF*α*, IL-6, and IL-17 were shown to promote tumor growth by modifying the function of stromal cells surrounding the tumor [63, 64]. TNF*α* produced by tumor cells or inflammatory cells in the tumor microenvironment can promote tumor cell survival through the induction of NF*κ*B-dependent antiapoptotic molecules [65]. TNF*α* was also shown to promote angiogenesis [66] and induce the expression of VEGF and HIF-1*α* in tumor cells [67]. IL-6 promotes angiogenesis and the expression of VEGF [68] through JAK2/STAT3 signaling [64] and the tumor promoting effects of IL-17 are in part mediated through upregulation of IL-6 [63, 64].

### 4.2. Angiogenesis and Modulation of the ECM

Angiogenesis is one of the hallmarks of the development of malignant neoplasias. Primary tumors of a certain size require the growth of new blood vessels in order to be supplied with nutrients and oxygen. Accordingly, at a size of 1-2 mm^3^, tumors alter their angiogenic phenotype and support continuous proliferation of endothelial cells. This “angiogenic switch” is activated by disturbed balance between endogenous pro- and antiangiogenic factors. It leads to the uncontrolled growth of blood vessels, mainly via stimulation of VEGF. Importantly, experimental* in vivo* models of angiogenesis have demonstrated that neutrophils affect neovascularization in the tissues [69]. Accordingly, Gr-1-mediated neutrophil depletion was found to significantly reduce tumor angiogenesis [70, 71]. Notably, in patients with myxofibrosarcoma, elevated numbers of neutrophils were observed in high-grade malignant tumors and this correlated positively with increased intratumoral microvessel density [72]. The mechanism by which tumor-associated neutrophils modulate tumor angiogenesis has not been fully elucidated. Activated neutrophils can release a variety of proteases that can degrade and remodel the ECM, a process that is crucial for angiogenesis. These cells have recently been shown to express high amounts of VEGF and MMP9 that is known to be responsible for initiation of the angiogenic switch and to support vessel growth in tumors [12]. MMP9 has been shown to have the most profound effects in mediating tumor angiogenesis [73]. Proteolysis of the ECM by this MMP releases such potent angiogenic factors such as vascular endothelial growth factor (VEGF) and FGF2 that are usually sequestered in an inactivated form to the ECM [74, 75]. MMP9 is also involved in the regulation of leukocytosis, for example, by potentiating proangiogenic and neutrophil attracting IL-8 expression [76] and by the release of hematopoietic progenitor cells from the bone marrow [77]. Huang et al. could show that MMP9-deficient mice display significantly reduced tumor microvessel density, compared with wild-type mice [78]. Neutrophil-derived MMP9 has also been shown to contribute to tumor angiogenesis and progression of squamous cell carcinoma [74]. Finally, Bv8, a potent proangiogenic factor, was shown to be upregulated in neutrophils in the context of cancer and to directly contribute to tumor angiogenesis and progression [79, 80].

### 4.3. Tumor Cell Dissemination

Metastasis is a highly complex process requiring tumor cell detachment from the primary tumor and migration to secondary target organs through the lymphatic or blood circulatory systems [81]. Neutrophils can exhibit both pro- and antimetastatic properties under certain conditions [82–85]. In prometastatic state neutrophils secrete soluble factors, including proteases and cytokines, that activate endothelium and parenchymal cells, leading to improvement of adhesion of circulating tumor cells in distant sites [74, 83, 86] and enhanced metastasis formation. Moreover, contact-dependent mechanisms, whereby neutrophils act as a bridge, tethering circulating tumor cells (CTCs) to target organ endothelium, have been described [87]. Such interaction is mediated by the binding of *β*2 integrins on neutrophils to ICAM-1 on tumor cells and was described for lung and liver metastasis model [84, 88]. In studies by Spicer et al. neutrophils promote cancer cell adhesion within liver sinusoids and their depletion before cancer cell inoculation resulted in decreased number of metastases in an intrasplenic model of liver metastasis [84]. Another interesting study showed that neutrophils can support lung metastasis development through physical interaction and anchoring of circulating tumor cells to endothelium [89]. It is not clear if this process supports tumor cell extravasation into target organ or neutrophils hold melanoma cells in the capillaries until they grow into a secondary tumor [89].

In addition to the mechanisms proposed thus far, novel aspects of neutrophil biology recently got attention as possible mechanism that contributes to cancer progression and metastasis. Recent studies suggest that NETs are able to trap tumor cells and depending on neutrophil activation such sequestered tumor cells can be destroyed by ROS that results in inhibition of metastasis formation [82] or be kept in place thus supporting early adhesion of tumor cells to distant organ sites and metastatic processes [90].

In the recent work of Wu et al. an inhibitory role of endogenous type I IFNs on neutrophil-mediated metastasis formation could be shown. The lack of endogenous type I IFNs drives neutrophils to prometastatic phenotype at least in two ways, supporting neutrophil migration and the formation of the premetastatic niche in the lung and inhibiting neutrophil cytotoxicity against tumor cells in circulation.

### 4.4. Formation of the Premetastatic Niche

Tumor induced changes in the microenvironment of distal organs make tissues more receptive to colonization of migrating tumor cells [91, 92]. Consequently, bone marrow derived cells, including neutrophils, are mobilized and accumulate in the future site of metastasis [93] where they participate in the formation of supportive metastatic microenvironment termed “premetastatic niche” [94–96]. These cells are recruited by Bv8, MMP9, S100A8, and S100A9 [6, 97] and this process seems to be strongly dependent on granulocyte colony-stimulating factor (G-CSF) [6].

Recent studies have shown that neutrophils make up the main cell population involved in formation of premetastatic niche [82]. This process seems to be enhanced by the absence of type I interferons that results in upregulation of CXCR2 expression on neutrophils from these mice. Moreover, prometastatic molecules like S100A8, S100A9, Bv8, and MMP9 are upregulated in lungs of Ifnar1^−/−^ mice. Both S100A8 and S100A9 are known to influence tumor cell proliferation, survival, and migration [97, 98] but also to stimulate migration and proliferation of neutrophils. Bv8, next to induction of tumor cell extravasation [6], increases neutrophil accumulation within premetastatic tissue. MMP9 is responsible for formation of leaky vasculature in the premetastatic lung [99] and support of tumor cells survival in this organ [100].

### 4.5. Recruitment of Other Cells and Immune Evasion

The immune regulatory functions of neutrophils are recently getting growing attention. Interactions between neutrophils and other immune cells obviously are regulating many inflammatory processes, including tumorigenesis. There is evidence that activated neutrophils can interact with T-cells in dichotomous ways. Several studies have shown that neutrophils can present antigens and provide accessory signals for T-cell activation [101–103]. Other studies have suggested that neutrophils can suppress antigen-nonspecific T-cell proliferation [104, 105]. The suppressive function of granulocytic cells in cancer patients has generally been attributed to a circulating low-density granulocytic myeloid derived suppressor cell (G-MDSC) population [60–62]. However, there is some uncertainty about whether G-MDSCs do exist in humans. In mice this heterogeneous group of cells consists mainly of immature neutrophils and monocytes.

Neutrophil-mediated T-cell suppression requires arginase 1 or ROS [105–107]. In humans with metastatic cancer disease, arginase 1-mediated suppression of lymphocytes was reported [108, 109]. Lately, mature blood neutrophil subset was shown to suppress T-cell activation in cancer [8] and during severe inflammation [104]. This suppression requires release of H_2_O_2_ into the immunological synapse in a Mac-1 (CD11b/CD18) dependent manner.

Very recent studies show that neutrophils cooperate with *γδ* T-cells in promotion of breast cancer metastasis [110]. Neutrophil depletion in the highly aggressive metastatic breast cancer mouse model KEP results in significant reduction of both spontaneous pulmonary and lymph node metastasis [110]. Moreover, combined depletion of both neutrophils and CD8^+^ cells results in inhibition of metastasis formation, implicating cooperation of these cells during this process.

## 5. Recruitment of Neutrophils into Tumor and Premetastatic Sites

Neutrophils make up substantial population of cells infiltrating tumors and premetastatic niche, in mice and human [12, 15, 111]. Many cell subtypes, including tumor cells, produce chemokines that attract neutrophils, for example, CXCL1 or CXCL2.

### 5.1. Factors That Mediate Neutrophil Recruitment

The migration of neutrophils into solid tumors depends on chemotactic factors. There are several chemotactic factors that may stimulate the migration of neutrophils, but the most potent are members of the CXCL chemokine family. Human CXCL8 (IL-8) is one of the best studied neutrophil chemoattractants with respect to tumor biology and is overexpressed in different human carcinomas and tumor cell lines including breast, colon, cervical, lung, brain, prostate, ovarian, and renal cell carcinomas, acute myelogenous and B-cell lymphocytic leukemia, melanoma, and Hodgkin's disease [112]. Importantly, both stromal and tumor cells can produce CXCL8. Other human chemokines such as CCL3 (MIP-1*α*) and CXCL6 (huGCP-2) or murine chemokines CXCL1, CXCL2, and CXCL5 are potent chemoattractants and activators for neutrophils [12] and are produced by many tumors [113–116]. Recent study on hepatocellular carcinoma indicated importance of CXCL16 and its receptor CXCR6 in neutrophil recruitment and tumor progression, due to its ability to stimulate tumor cells to release CXCL8. Another recent study shows that human metastatic melanoma cells entrapped in the lungs secrete IL-8 to attract neutrophils, which promotes tumor cell tethering to the vascular endothelium. Prolonged cell retention in the lungs facilitated transendothelial migration and metastasis development [89]. Experiments have shown that inhibition of neutrophil migration, for example, by blocking of chemokine receptor CXCR2 or CXCR2^−/−^ in mice, leads to reduced tumor angiogenesis and growth in B16F10 melanoma [14, 117] and Lewis lung carcinoma model [107]. Inhibited myeloid cell infiltration due to the loss of CXCR2 was also shown to be responsible for significantly suppressed chronic colonic inflammation and colitis-associated tumorigenesis [118].

A number of additional mediators might serve as chemoattractants for neutrophil recruitment to the tumor tissue. It has been shown that bioactive lipids, such as sphingosine-1-phosphate (S1P), could promote neutrophil activation and chemotaxis [119, 120]. Similarly, the hypoxia-inducible factor 1-*α* and its downstream products like CXCL12, VEGF, or MMP9 are involved in recruitment and retention of neutrophils in angiogenic environments [12, 121]. VEGF, in addition to its proangiogenic role during tumor growth, is also capable of inducing neutrophil adhesion to postcapillary venules followed by homing to tissues of its high expression, for example, tumor or premetastatic niche [122, 123].

Recent studies suggest that the myeloid-related proteins (MRPs) are also involved in neutrophil migration. The MRPs S100A8 and S100A9 are strongly expressed by tumors and in the premetastatic niche and act as strong chemoattractants for neutrophils into these sites [82, 97, 124]. However, the exact mechanism of MRPs mediated neutrophil mobilization is not clear and still needs to be investigated.

### 5.2. Survival of Neutrophils in Tumor Microenvironment

Due to their proinflammatory functions and potential toxicity against host tissue, the neutrophil life span is strictly regulated [125]. In the absence of inflammatory stimuli, neutrophils are removed from circulation shortly after their mobilization from the bone marrow, mainly by apoptosis. Importantly, several proinflammatory cytokines have been shown to influence the longevity of neutrophils [126]. Recent observations of Andzinski et al. [13] show that the life span of tumor-associated neutrophils is remarkably prolonged in tumor-bearing IFN-*β* deficient (Ifnb1^−/−^) mice, compared to wild-type controls. This is apparently due to the fact that IFN-*β* is able to influence both the extrinsic and the intrinsic apoptosis pathways of neutrophilic granulocytes. Lower expression of Fas, reactive oxygen species, active Caspases 3 and 9, as well as a change in expression pattern of proapoptotic and antiapoptotic members of the Bcl-2 family and the major apoptosome constituent Apaf-1, is observed under such conditions. The death receptor Fas on neutrophils has been shown to be involved in spontaneous extrinsic cell death signaling [127]. Fas has been shown to play a role in type I IFN-induced apoptosis in several types of neoplasias such as melanoma, multiple myeloma, and chronic myeloid leukemia cells [128, 129].

ROS production by neutrophils might also play an important role in regulation of life span of neutrophils. For example, a delayed spontaneous apoptosis was shown in patients deficient for NADPH oxidase [130, 131]. It has also been shown that hypoxia or pharmacological inhibition of NADPH oxidase and hydrogen peroxide scavengers decreases the rate of neutrophil apoptosis [132]. Recent data indicate that spontaneous production of ROS is diminished in the absence of endogenous IFN-*β*, potentially contributing to the delayed apoptosis of tumor infiltrating neutrophils in Ifnb1^−/−^ mice [13]. G-CSF is one of the major survival factors of neutrophilic granulocytes and has been reported to reduce Bax expression and redistribution [133] and restore its phosphorylation status thus leading to its inactivation. This mechanism is responsible for G-CSF-mediated repression of Caspase activation [134]. Regulation of G-CSF expression is responsible for altered neutrophils survival in tumors.

## 6. Concluding Remarks

Neutrophil function in cancer has long been a matter of debate as these cells were shown to possess a range of tumor promoting as well as tumor limiting properties. We propose that these conflicting observations stem from the fact that neutrophils are not a homogeneous population of cells. Neutrophil heterogeneity stems from two facts that are not mutually exclusive and have to do with the changes in the chemokine milieu in the context of cancer: The first is the fact that neutrophils are highly responsive to cues in their microenvironment and may adopt a protumor phenotype in certain conditions and an antitumor phenotype in others. The second is the fact that there are distinct neutrophil subsets which differ in their contribution in the context of cancer. Together, these observations support the notion that neutrophil function in cancer may be dictated in a context dependent fashion (Figure 1). These observations also identify potential elements which may be therapeutically targeted to enhance antitumor neutrophil activity while limiting their protumor properties.
